# Accelerating epistasis analysis in human genetics with consumer graphics hardware

**DOI:** 10.1186/1756-0500-2-149

**Published:** 2009-07-24

**Authors:** Nicholas A Sinnott-Armstrong, Casey S Greene, Fabio Cancare, Jason H Moore

**Affiliations:** 1 Computational Genetics Lab, Department of Genetics, Norris-Cotton Cancer Center, Dartmouth Medical School, Lebanon, NH, USA; 2 Dipartimento di Elettronica e Informazione, Politecnico di Milano, Milano, Italia

## Abstract

**Background:**

Human geneticists are now capable of measuring more than one million DNA sequence variations from across the human genome. The new challenge is to develop computationally feasible methods capable of analyzing these data for associations with common human disease, particularly in the context of epistasis. Epistasis describes the situation where multiple genes interact in a complex non-linear manner to determine an individual's disease risk and is thought to be ubiquitous for common diseases. Multifactor Dimensionality Reduction (MDR) is an algorithm capable of detecting epistasis. An exhaustive analysis with MDR is often computationally expensive, particularly for high order interactions. This challenge has previously been met with parallel computation and expensive hardware. The option we examine here exploits commodity hardware designed for computer graphics. In modern computers Graphics Processing Units (GPUs) have more memory bandwidth and computational capability than Central Processing Units (CPUs) and are well suited to this problem. Advances in the video game industry have led to an economy of scale creating a situation where these powerful components are readily available at very low cost. Here we implement and evaluate the performance of the MDR algorithm on GPUs. Of primary interest are the time required for an epistasis analysis and the price to performance ratio of available solutions.

**Findings:**

We found that using MDR on GPUs consistently increased performance per machine over both a feature rich Java software package and a C++ cluster implementation. The performance of a GPU workstation running a GPU implementation reduces computation time by a factor of 160 compared to an 8-core workstation running the Java implementation on CPUs. This GPU workstation performs similarly to 150 cores running an optimized C++ implementation on a Beowulf cluster. Furthermore this GPU system provides extremely cost effective performance while leaving the CPU available for other tasks. The GPU workstation containing three GPUs costs $2000 while obtaining similar performance on a Beowulf cluster requires 150 CPU cores which, including the added infrastructure and support cost of the cluster system, cost approximately $82,500.

**Conclusion:**

Graphics hardware based computing provides a cost effective means to perform genetic analysis of epistasis using MDR on large datasets without the infrastructure of a computing cluster.

## Background

Advances in chip-based genotyping technology have made routine the measurement of one million DNA sequence variations. Human geneticists are no longer limited by the measurement of genetic variations, and instead are limited by the analysis of these variations. This is especially true when epistasis is considered. Epistasis is characterized by interaction between variations. In this situation, variations must be analyzed in the context of other variations to detect and characterize gene-disease associations. Epistasis likely forms the genetic basis of many common human diseases [1]. Multifactor dimensionality reduction (MDR) is an generic algorithm capable of detecting epistasis, but an exhaustive analysis is combinatorial in complexity [2].

Assuming a modern study of one million DNA sequence variations, there are 5.0 × 10^11 ^possible pairwise interactions. This number grows to 3.3 × 10^17 ^for three-way interactions. Analyses of high order interactions between three or more genes quickly approach the limits of current technology. Approaches have been developed which exploit statistical pre-processing to choose either a subset of DNA sequence variations to exhaustively evaluate or a subset of potential interactions to examine [3-8]. Even approaches examining a small fraction (i.e. 1% of potential interactions) are computationally expensive on datasets of this size and can benefit from greater performance. Here we examine whether the modern Graphics Processing Unit (GPU), a massively parallel hardware platform, provides performance benefits and cost effectiveness. Advances in performance will allow researchers to more fully examine these genome-wide data for the epistatic interactions believed to underlie common human diseases.

### Multifactor Dimensionality Reduction (MDR)

The MDR algorithm, developed by Ritchie et al. [2], is currently provided in an open source package. The MDR software package uses the Java programming language and features a powerful GUI and a variety of preprocessing, expert knowledge, and visualization extensions. Here we compare the performance of the GPU solution against this software package, as well as against an optimized C++ version designed to run on clusters of computers.

The MDR algorithm is conceptually simple. Given a set of SNPs, a threshold *T*, and the case-control status *P*, a new attribute *G *is constructed. *G *is considered low risk if the ratio of cases to controls given the SNPs is less than *T *and high risk if the ratio is greater than *T*. In this way, the multidimensional SNP data is captured as a single-dimensional attribute *G*. The combination of an easy to use interface and an effective design have led to the use of the MDR package in a number of studies [7,9]. Here we develop an implementation of MDR capable of running on graphics processing units (GPUs) using the NVIDIA Compute Unified Device Architecture (CUDA) framework.

### The Graphics Processing Unit

In modern computers capable of running graphics intensive applications, the memory bandwidth available to GPUs is far greater than to other components. High performance graphics cards, such as NVIDIA Corporation's GTX 280 that we use here, have more than 10 times as much memory bandwidth available to them as modern CPUs [10,11]. The GPU's order of magnitude advantage in memory bandwidth greatly increases performance for large datasets.

On a typical consumer computer system, video games or other applications using 3D graphics are the most data-intensive applications. A single screen can contain millions or billions of triangles that need to be processed with lighting constants and shape deformations and then displayed on the screen. Recently game developers have released games with sophisticated graphics [12,13], which are driving improvement in GPU technology. The photo-realistic details demanded by the consumer market have forced GPU manufacturers to develop faster hardware. GPU manufacturers now run code in parallel across multiple cores, thus increasing the speed with which the overall jobs complete. NVIDIA GTX 280 has 240 processors [10], each doing its own work, which run in parallel and greatly enhance rendering performance.

Many tasks can benefit from parallel execution through the large number of cores available [14-16]. The parallel architecture provides more flexibility in the rendering pipeline, offloading work of graphics design to game producers. Coincidentally, this flexibility also enables applications other than games to exploit GPUs. Their architecture, speed, and low price make GPUs a viable alternative for high performance computation.

While GPUs are very efficient for many scientific applications, they are not well suited for all tasks. GPUs provide data level parallelism, so they work well for parallelizing tasks which depend on applying a small number of steps to a large amount of data. When algorithms depend on applying many interdependent operations to small amounts of data the GPU is unlikely to greatly increase performance. MDR can be implemented in an iterative fashion that allows for efficient execution on GPUs.

## Findings

MDR on GPUs performs better on lower cost hardware than MDR on CPUs. We find that a single GPU is capable of outperforming an eight CPU core workstation running a Java version by a factor of approximately 60 and that its performance falls between that of a 32 core and 80 core CPU cluster running a C++ implementation. A GPU workstation containing three GPUs is capable of performance equal to approximately 150 clustered CPU cores running a C++ version and it outperforms the Java version running on an eight core workstation by a factor of 160.

### Performance Results

We specifically compare MDR on GPUs to two other frameworks. Performance results are shown in Table 1. First, we compare the performance to the fully featured user friendly GUI version implemented in Java running on a single machine. This situation represents how many researchers approach an MDR analysis when the infrastructure of a computing cluster is unavailable. We examine the performance given two different configurations, an 8-core workstation (with eight Xeon X5472 cores at 3.0 Ghz) with 64 GB of RAM and a 4-core workstation (with four Xeon X5365 cores at 3.0 Ghz) with 4 GB of RAM. Our simulated benchmark dataset contains 1600 individuals and 1000 SNPs. This dataset is similar to what would be seen in a modestly sized study. On this test dataset, the 11741.932 seconds taken by our highest end eight core workstation running the Java version is 160 times longer than the 72.633 seconds taken on average by the three GPU implementation. The time taken by two GPUs is 102.250 seconds, about three halves of the time for three GPUs and still far below the time taken by the Java version on the highest end workstation. Even using just one GPU, where the time increases to 199.260 seconds, the GPU workstation outperforms the eight core workstation by more than a factor of 60. The GPU implementation of the MDR algorithm greatly and consistently outperforms the CPU Java implementation on both four and eight core workstations. As Java sacrifices speed for portability, this speed increase is not unexpected, but it is still informative because most users perform these analyses on a single workstation.

**Table 1:** Execution Time

Data set size	Host	Time 1	Time 2	Time 3	Avg time	Std. dev time
1600 × 1000	3 GPU	72.639	72.649	72.611	72.633	0.016
1600 × 1000	2 GPU	102.243	102.228	102.276	102.249	0.020
1600 × 1000	1 GPU	193.319	193.243	193.207	193.256	0.047

1600 × 1000	8 core (Java)	11676.730	13782.761	9766.305	11741.932	1640.359
1600 × 1000	4 core (Java)	18656.208	19251.764	14188.972	17365.648	2259.369

1600 × 1000	4 core (C++; cluster)	2664.016	2198.369	2306.662	2389.682	198.957
1600 × 1000	16 core (C++; cluster)	669.783	668.732	660.090	666.202	4.343
1600 × 1000	32 core (C++; cluster)	335.186	301.622	359.725	332.178	23.816
1600 × 1000	80 core (C++; cluster)	129.589	129.171	129.815	129.525	0.267
1600 × 1000	150 core (C++; cluster)	67.660	69.814	69.938	69.137	1.046

The execution times of various hosts calculating all three-way interactions of a particular data set. The times are all as reported by the Unix time(1) command.

**Figure 1:**
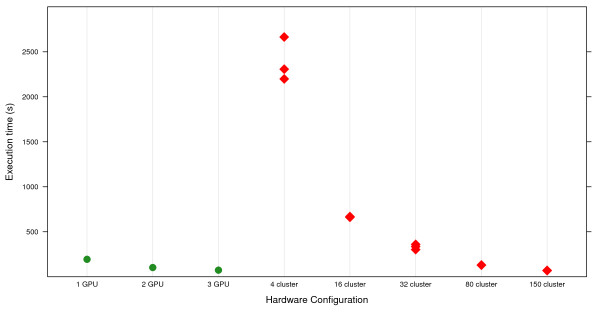
**Execution time comparison**. A comparison of the execution times of three-way detection in a 1600 × 1000 data set for GPU and cluster MDR platforms. The Java implementation running on workstations is not included because it performs relatively poorly and inclusion makes distinguishing the performance of the GPU and cluster implementations difficult.

Second we compare the performance on GPUs to a C++ version of MDR running on a Beowulf cluster of computers. We compare our GPU benchmarks to results obtained using 4, 16, 32, 80, or 150 cores on a cluster. These results are shown in Table 1. A workstation using a single GPU outperforms 32 cores on the cluster and underperforms 80 cores on the cluster. A workstation using three GPUs performs similarly to 150 cores on the cluster. This comparison is important because some users are interested in high performance computing but do not have access to or infrastructure for a cluster of compute nodes. Here we show that a single workstation can perform an amount of work similar to a reasonably sized cluster [see Figure 1].

### Cost

Not only is the GPU solution faster than using standard CPUs, but it is also less expensive. The three GPU workstation [see Additional File 1] costs approximately $2200. The GPU implementation is not CPU bound and thus an inexpensive CPU can be used. This compares very favorably to the 4-core workstation, which costs about $5000, and the 8-core workstation, which costs about $13750. The cost of 150 cores on the Beowulf cluster, including infrastructure and support, is approximately $82,500, which provides performance similar to that obtained with three GPUs. The GPU workstation only requires infrastructure and support similar to a standard workstation. The researchers included in their GPU workstation the option of upgrading to four GPUs, but if this possibility is not considered, then the cost can be lowered to $1700 [see Additional File 2]. The price to performance ratio, shown in Table 2, thus exceeds 1000:1 when the GPU implementation replaces multi-core CPUs directly. Performance of the GPU approach can be increased by adding more GPUs to each workstation or with additional GPU machines in a cluster environment, although this approach decreases some of the infrastructure benefits.

**Table 2:** Server cost and price to performance ratio

Solution	Cost	Average time	Price to performance
8-core CPU	13750	11742.000	1.0000
4-core CPU	5000	17366.000	1.8594

4 core cluster	2200	2389.700	30.710
16 core cluster	8800	666.200	27.540
32 core cluster	17600	332.180	27.616
80 core cluster	44000	129.530	28.328
150 core cluster	82500	69.137	28.306

1 GPU	970.94	193.260	860.42
2 GPU	1320.93	102.250	1195.4
3 GPU	1670.92	72.633	1330.3

This is a comparison between the different solutions, taking into account their cost and scaled for the price to performance ratio of the 8-core server running Java. The cluster costs derive from a base cost of $4400 for an eight core node.

## Conclusion

The implementation and analysis of MDR on GPUs has shown that general purpose GPU computing is well suited to MDR and other algorithms which rely on processing large amounts of mutually independent data. Consumer demand for very high performance graphics hardware has lowered the cost of high-performance GPU systems for scientific research to a level far below the cost of similar performance CPU systems. Researchers performing epistasis analysis using MDR should examine their requirements and determine whether CPUs or GPUs provide a more appropriate framework for analysis. Individuals performing analysis of large datasets or permutation testing would benefit most from a GPU machine or set of machines.

## Methods

### Libraries and Dependencies

The MDR implementation on GPUs [17] is based around the NVIDIA CUDA framework [18] and Python programming language [19], with a binding called PyCUDA [20]. This model, along with the pp library for parallel execution [21], allows for distributed, networked, high-performance clusters of GPUs that can simultaneously perform a single task. The Numpy library [22] is used for efficient manipulation of the data arrays.

### GPU Implementation

In order to understand the details of the GPU implementation of MDR algorithm, one must first understand the GPU execution model. The current (8x00 series and above) NVIDIA GPU is best modeled as, "a set of SIMD execution units with high bandwidth shared memory and a tiered execution hierarchy" [23]. The basic unit of execution is the *kernel*, which is a block of code which is executed by a group of threads in parallel. The block and grid constructions easily support multiple level parallelism. The GPU implementation of MDR uses this tiered execution architecture to its advantage by running a number of threads in parallel.

Each block in the GPU implementation is responsible for running a single triplet (or pair in the two-way implementation) of columns. The threads in the block work in parallel using the bucket method similar to that described in [24] to quickly determine the accuracy on a per column basis. The grid is responsible for running these column accuracy calculations and additionally runs a reduction which finds the best solution. Once the structure of the block and thread hierarchy has been defined, the MDR algorithm must be considered. As stated above, the main steps that the MDR algorithm follows are:

1. Across every set of two (or three, ...) genotype attributes selected, sum the case/control statuses present for each combination of attributes. This step is called "bucketing."

2. Determine an estimated status marker for each bucket by labeling the bucket as high risk if it contains more cases than expected based on the proportion of cases in the dataset and low risk otherwise.

3. Find the balanced accuracy of all the buckets by looping over every genotype's subjects again and finding the sensitivity and specificity of the estimated status markers calculated above. The arithmetic mean of the sensitivity and specificity is called the balanced accuracy.

4. Find the genotype combination with the highest balanced accuracy. This genotype combination is the one most predictive of the case/control status of individuals in the dataset.

Next, each step must be broken down into dependencies. For example, the accuracy calculation first needs to have an estimated status marker calculation, which in turn depends on the initial bucketing but no information must be accessed outside of a single combination of genotypes. If arrays need to be shared between threads, the CUDA memory architecture should be exploited to either use constant memory (if the arrays do not change) or shared memory (if they do).

One of the main difficulties with writing CUDA code is organizing the memory to maintain high resource utilization and efficiency. There are four main memory spaces the authors used to accomplish this goal: constant memory, global memory, shared memory, and registers. Texture memory and local memory were evaluated as well for storing genotype data, but they were found to slow down computation.

Constant memory, as its name implies, stores constants – values which cannot be changed by kernels running on the GPU. This makes them limited to lookup tables and similar data structures. In the GPU implementation of MDR, they are used to store the phenotypes of all the individuals. Because constant memory is cached and localized, phenotypes are accessed in linear order to ensure spatial locality and cache coherency.

Global memory is the actual RAM which resides on the graphics card, attached via the printed circuit board to the GPU itself. It is slow, but if used correctly can still yield acceptable results. The implementation holds the genotype array directly in global memory. Since only two lookups are used per attribute per run, the overhead is minimal. The authors tried other solutions, such as caching global memory in shared memory or using the aforementioned texture memory, but none of these were as fast in a variety of situations as the pure global reads. It should be noted that the authors run on the GTX 200 architecture, which supports limited autocoalescing of global reads.

Shared memory is a small RAM buffer (16 K) which can be accessed by all threads within a block. It has a variety of uses (caching, intermediate results, ...), and many of these are used extensively. Most importantly, the parallel reductions which are prevalent in the program design and the buckets which form the main storage component of the program both act on and reside in shared memory.

Registers are storage locations which are thread-local, so only a single thread in a block can access their value and they are unique across threads. This makes registers critical for keeping track of which values an individual thread should compute and also for storing results of global reads.

Finally, it is important to note that Python has a number of restrictions which relate to running code in parallel (actually executing two pieces of code simultaneously). Most limiting is the Global Interpreter Lock [25], in which limits access to I/O resources to only one thread at a time. Practically, this means that only one instance of PyCUDA can run in each Python instance, so only one GPU can be utilized per execution, even though the GPU implementation does not tax the CPU. While there are ways around this single-active-GPU-per-process limitation (by saving and restoring PyCUDA contexts [26]), the better solution is to use an external library to run two Python instances at the same time from the same file. We chose to use the pp library for parallel Python execution [21], as it allows for seamless parallel execution not only across cores but also across machines. It would be simple to add any new workstations purchased to the list of servers and enable execution across all of the machines simultaneously.

## Competing interests

The authors declare that they have no competing interests.

## Authors' contributions

NAS-A implemented the MDR solution for GPUs and prepared the manuscript. CSG assisted with the implementation and prepared the manuscript. FC assisted with the optimization of the GPU code. JHM assisted with the implementation and prepared the manuscript.

## Supplementary Material

Additional File 1 **3 GPU Server sample BOM**. This is a sample bill of materials for a GPU Server which can handle up to three GPUs. Cost is estimated. Almost identical performance should be achieved for much lower overall cost due to only using the components necessary for running three GPUs, not four. Click here for file

Additional File 2 **Upgradeable 3 GPU Server sample BOM**. This is the bill of materials for our GPU Server which can handle up to four GPUs (if an additional power supply is added or the Thermaltake Toughpower 1500 W 230 V power supply is used) and is configured with three GPUs. Cost is estimated based on prices from February 2009. Click here for file
