# Supplementary material for: Accelerating epistasis analysis in human genetics with consumer graphics hardware
Source: BMC Res Notes. 2009 Jul 24;2:149. doi: 10.1186/1756-0500-2-149 (PMC2732631; doi:10.1186/1756-0500-2-149)
Supplement: Additional File 1 — 3 GPU Server sample BOM. This is a sample bill of materials for a GPU Server which can handle up to three GPUs. Cost is estimated. Almost identical performance should be achieved for much lower overall cost due to only using the components necessary for running three GPUs, not four. [file 1756-0500-2-149-S1.pdf]

Item	Number	Cost (ea.)	Total Cost
AMD Toliman 8450	1	79.00	79.00
BFG Tech BFGEGTX2801024OCXE GTX 280 1GB	3	349.99	1049.97
Antec Nine Hundred Mid Tower	1	99.99	99.99
MSI K9A2 Platinum	1	149.99	149.99
HIPER Type R II HPU-5K880 880W	1	179.99	179.99
OCZ Platinum 4GB (2 x 2GB) DDR2 1066	1	63.99	63.99
250GB WD2500AAJS 7200 RPM Hard Drive	1	47.99	47.99
TOTAL	9		1670.92
