# Supplementary material for: Accelerating epistasis analysis in human genetics with consumer graphics hardware
Source: BMC Res Notes. 2009 Jul 24;2:149. doi: 10.1186/1756-0500-2-149 (PMC2732631; doi:10.1186/1756-0500-2-149)
Supplement: Additional File 2 — Upgradeable 3 GPU Server sample BOM. This is the bill of materials for our GPU Server which can handle up to four GPUs (if an additional power supply is added or the Thermaltake Toughpower 1500 W 230 V power supply is used) and is configured with three GPUs. Cost is estimated based on prices from February 2009. [file 1756-0500-2-149-S2.pdf]

Item	Number	Cost (ea.)	Total Cost
AMD Phenom 9850 Black Edition	1	145.00	145.00
BFG Tech BFGEGTX2801024OCXE GTX 280 1GB	3	349.99	1049.97
LIAN LI PC-P80	1	379.99	379.99
MSI K9A2 Platinum	1	144.99	144.99
COOLER MASTER Real Power Pro 1250W	1	329.99	329.99
OCZ Platinum 4GB (2 x 2GB) DDR2 1066	1	63.99	63.99
250GB WD2500AAJS 7200 RPM Hard Drive	1	47.99	47.99
TOTAL	9		2161.92
